# LMNA functions as an oncogene in hepatocellular carcinoma by regulating the proliferation and migration ability

**DOI:** 10.1111/jcmm.15829

**Published:** 2020-09-08

**Authors:** Heng Liu, Dongming Li, Ling Zhou, Shuang Kan, Guozhang He, Kun Zhou, Liping Wang, Ming Chen, Wei Shu

**Affiliations:** ^1^ College of Biotechnology Guilin Medical University Guilin China; ^2^ College of Stomatology Guangxi Medical University Nanning China; ^3^ Department of Cell Biology and Genetics Guangxi Medical University Nanning China; ^4^ State Key Laboratory for the Chemistry and Molecular Engineering of Medicinal Resources School of Chemistry and Pharmacy Guangxi Normal University Guilin China; ^5^ Department of General Surgery The Third Affiliated Hospital of Southern Medical University Guangzhou China

**Keywords:** CRISPR/Cas9, Hepatocellular carcinoma (HCC), LMNA, P16

## Abstract

The role of the LMNA gene in the development and progression of hepatocellular carcinoma (HCC) and the associated molecular mechanism is not yet clear. Therefore, the purpose of this study was to evaluate the relationship between LMNA and HCC. LMNA gene expression in normal tissues and corresponding tumours was evaluated and the Kaplan–Meier survival analysis was performed. Next, the LMNA gene was knocked out in the 293T and HepG2 cell lines using the CRISPR/Cas9 technique. Subsequently, the proliferation, migration and colony formation rate of the two LMNA knockout cell lines were analysed. Finally, the molecular mechanism affecting the tumorigenesis due to the loss of the LMNA gene was evaluated. The results showed that the LMNA gene was abnormally expressed in many tumours, and the survival rate of the HCC patients with a high expression of the LMNA gene was significantly reduced compared with the rate in patients with a low LMNA expression. The knockout of the LMNA gene in the HCC cell line HepG2 resulted in a decreased tumorigenicity, up‐regulation of the P16 expression and down‐regulation of the CDK1 expression. These findings suggested that LMNA might function as an oncogene in HCC and provided a potential new target for the diagnosis and treatment of HCC.

## 1 INTRODUCTION

Liver cancer is a relatively common cancer worldwide, and it is mainly divided into primary liver cancer and metastatic liver cancer. Primary liver cancer is currently the sixth most common tumour in the world and the third most common cause of cancer‐related death.^1^, ^2^ The latest reports show that primary liver cancer is the fourth most common cancer in China.^3^ Hepatocellular carcinoma (HCC) is a type of primary liver cancer. In the early stage of liver cancer, the clinical symptoms of the patients are not evident, although the disease develops quite rapidly. Generally, a correct diagnosis is reached when the liver cancer is in its late stage, thus becoming a greater threat to the life and health of patients, and resulting in a higher probability of death.^4^, ^5^ Due to its complexity, recurrence, metastasis and heterogeneity after surgical resection, HCC is one of the most deadly cancers even after surgical resection.^6^

Like other cancers, HCC is also characterized by an abnormal gene expression. The LMNA gene encodes the two main isoforms lamin A and lamin C. Lamins are structural proteins forming the nuclear lamina, which is the inner nuclear membrane determining the nuclear shape and size. Three types of lamins have been previously described in mammal cells, such as A, B and C.^7^ Lamin B1 and vimentin were the main overexpressed proteins in liver cancer tissues.^8^ Thus, Lamin B1 and vimentin in the blood could be used as novel biomarkers for early HCC and can be detected by non‐invasive methods.^9^ Lamin A expression varies in a variety of tumour cells. Its expression decreases in breast, prostate, colon, ovarian, gastric and endometrial cancer, leading to a reduction in overall survival and an increase in the number of metastatic sites and tumour recurrence.^10^, ^11^, ^12^, ^13^ In contrast, some studies revealed a link between increased lamin A expression and the development and progression of colorectal cancer, prostate cancer and ovarian cancer.^14^, ^15^, ^16^ However, the role of the LMNA gene in the development and progression of HCC and the associated molecular mechanism remains unknown.

Therefore, the purpose of this study was to evaluate the relationship between LMNA and HCC. The LMNA gene was knocked out in 293T and HepG2 cell lines by the CRISPR/Cas9 technology. Subsequently, the proliferation, migration and colony formation rate of the two LMNA knockout cell lines were analysed, and the tumorigenicity in vivo was tested in a subcutaneous tumour mouse model. Finally, the molecular mechanism affecting the tumorigenesis due to the loss of the LMNA gene in the 293T and HepG2 cells was explored.

## 2 MATERIAL AND METHODS

### 2.1 Ethical guidelines

All the experiments in this study were performed according to the guidelines of the Experimentation Ethics Committee of the Guilin Medical University, China (approval No. 2019‐0008).

### 2.2 Bioinformatic

Data related to the LMNA gene expression in various normal tissues and corresponding tumours were collected from the Proteomics DB, Max QB and MOPED databases. The Kaplan–Meier survival analysis was performed by the Kaplan–Meier plot (http://kmplot.com/analysis).

### 2.3 Cell lines and culture conditions

The human liver cancer cell line (HepG2) and HEK 293 kidney cell line expressing a mutant version of the SV40 large T antigen (293T) were stored in our laboratory. Cells were routinely cultured in Dulbecco's Modified Eagle's Medium (DMEM; Gibco, CA, USA) supplemented with 10% foetal calf serum (Gibco, CA, USA) and incubated at 37°C in a 5% CO_2_ atmosphere. They were cultured until reaching 50% to 80% confluence before the next passage or further experiments.

### 2.4 gRNA design

The gRNA was designed using the Massachusetts Institute of Technology's CRISPR Design software (http://crispr.mit.edu/), and the designed primers were confirmed by the Primer‐Blast tool of NCBI. Four gRNAs were designed as follows: FO15′‐TTCCGCCAGCAGCCGCCGGC‐3′, RO15′‐GCCGGCGGCTGCTGGCGGAA‐3′, FO25′‐AGCGGGAGATGGCCGAGATG‐3′, RO25′‐CATCTCGGCCATCTCCCGCT‐3′, FO3 5′‐CACGCAGCTCCTGGAAGGGT‐3′, RO35′‐ACCCTTCCAGGAGCTGCGTG‐3′, FO45′‐GCGCCGTCATGAGACCCGAC‐3′, RO45′‐GTCGGGTCTCATGACGGCGC −3′.

### 2.5 CRISPER/Cas9 technique

The resultant gRNA justice chain and antisense chain were renatured to form a double chain. The reaction system was the following: 1 μl of each positive and antisense chain, T4 linkase buffer (10×) 1 μl l, T4 polynucleotide kinase (PNK) 0.5 μl, double distilled water 6.5 μl, 95°C for 5 minutes, and left to cool down to 16°C for 10 minutes. Then, BbsI endonuclease was used to shear PX459 at 4°C overnight, and the plasmid, after shearing, was recovered by electrophoresis. The oligonucleotide double chain and the post‐shear plasmid were recombined using T4 ligase. The reaction system was as follows: PX459 plasmid 50 ng, oligonucleotide double chain 2 μl, T4 connectase 1.5 μl, T4 connectase buffer solution 1.5 μl, double evaporation of the water until reaching 15 μl. The reaction procedure was as follows: after combining the oligonucleotide double chain and the T4 connectase at 16°C for 10 minutes, the reaction system was left overnight at 4°C. The recombined carrier suspension product of 5 ~ 10 μl was added to the 50 μl DH5a sensor cells, which were lightly blended and bathed in ice for 30 minutes, heated at 42°C for 90 seconds, left on ice for 2 minutes, and then directly coated on an ampicillin resistant LB plate. The reaction system was incubated overnight at 37°C, 3 ~ 5 white bacterial colonies were selected for culture, and plasmid DNA was extracted for sequencing verification and amplification. The amplified plasmid DNA was collected and stored at −20°C. The sequencing primers were the following: upstream primer 5'‐GAGGGCCTATTTCCCATGAT‐3', downstream primer 5'‐GGGCGTACTTGGCATATGAT‐3'. The primers were synthesized by the Shanghai sangon biological engineering technology service co., LTD.

Two million cells were added to each well in a 6‐well plate and washed with PBS twice after 12 hours. The Lipofectamine 3000 Transfection reagent (10 μl, Invitrogen, USA) was mixed with 250 μl bovine free media, and left at room temperature for 5 minutes. Meanwhile, 5 μl recombinant plasmid (or exogenous LMNA expressing plasmid) was mixed with 250 μl bovine free media, and left at room temperature for 5 minutes. Then, the two liquids were thoroughly mixed and left at room temperature for 20 minutes. Finally, the mixed liquid was transferred into a culture plate. The complete culture medium was changed at 6‐8 hours after transfection, fresh complete culture medium was replaced at 24 hours after transfection, and puromycin was added for screening (working concentration of 293T was 2.0 μg/mL, and that of HepG2 was 1.5 μg/mL). The screened cells were cultured for subsequent experiments.

### 2.6 Immunofluorescence

The cells were immersed in PBS and seeded in glass slides at a concentration of 1 × 10^4^/ml. The slides were fixed in 4% paraformaldehyde for 15 minutes, then immersed in PBS 3 times for 3 minutes each time, and subsequently in 0.5% Triton X‐100 in PBS at room temperature for 20 minutes. Next, the cells were blocked with 5% skim milk powder in PBST for 30 minutes at room temperature, and the following diluted primary antibodies were added: anti‐Lamin B, BOSTER, China; anti‐β‐actin, Abcam, USA; anti‐Lamin A, Abcam, USA. The antibodies used were rabbit anti‐human and diluted at a concentration of 1:200. The cells were incubated overnight at 4°C and subsequently with the fluorescent secondary antibody (1:100, mouse anti‐rabbit antibody, Abcam, USA) for 1 hour at room temperature. Finally, the slides were washed in PBST 3 times for 3 minutes each and observed under a fluorescence microscope.

### 2.7 Western blot

RIPA lysis buffer (plus PMSF) was used to lyse the cells to extract the total proteins, which were separated by a polyacrylamide gel electrophoresis and transferred into the PVDF membrane. After membrane blocking, the corresponding primary antibodies (1:1000, rabbit anti‐human, anti‐Lamin A/Lamin C/β‐actin, Abcam, USA, and anti‐P16/CDK1/MMP2/MMP9, BOSTER, China) were added to the membrane that was incubated overnight at 4°C. Next, the secondary antibody (1:8000, mouse anti‐rabbit antibody, Abcam, USA) was added to the membrane that was incubated at room temperature for 1 hour. Finally, the bands were imaged using Gel imaging system.

### 2.8 CCK‐8 cell growth assay

The 293T and HepG2 wild‐type and knockout cell lines were trypsinized, seeded into 96‐well plates at a concentration of 1500 cells per well in triplicate, and cultured at 37°C. Cell counting kit‐8 (KeyGen, China) was added after 18 hours and incubated for 1 hour. The absorbance was measured at 450 nm every other day until one of the cells reached the confluence of more than 70% to stop the cell proliferation experiment.

### 2.9 Cell cycle detection

The cells in the logarithmic growth phase were seeded in a 6‐well plate at a density of 1 × 10^6^ cells/mL in 2 mL medium and in a 24‐well plate in 1 mL medium, and the cells were collected after 24 hours. After centrifugation at 800 rpm for 5 minutes, the supernatant was discarded, and the cell pellet was collected, washed twice with pre‐cooled PBS, and with pre‐cooled with 75% ethanol. Next, the cells were fixed at 4°C for more than 4 hours. After centrifugation at 1500 rpm for 5 minutes, the supernatant was discarded, the cells were washed once with 3 mL PBS, and finally 400 uL ethidium bromide (PI, 50 µg/mL), and 100 µl RNase A (100 µg/mL) (KeyGen, China) were added, and the cells were and incubated at 4°C for 30 minutes in the dark. Flow cytometry was used to detect 20 000‐30 000 cells by a standard procedure, and the results were analysed by the cell cycle FACS software.

### 2.10 Apoptosis detection

The cells were collected, washed once with PBS, centrifuged at 1000 r/min for 5 minutes, and the supernatant was discarded. According to the kit instructions, each sample (293T‐WT, 293T‐KO, HepG2‐WT, HepG2‐KO) was resuspended in 100 μL binding buffer and then Annexin V‐FITC (5 μL/tube) and PI staining solution (5 μL/tube) (KeyGen, China) were added and incubated for 10 minutes in the dark. Next, 400 μL binding buffer was added to each tube, tubes were gently mixed, and flow cytometry analysis was performed within 1 hours.

### 2.11 Wound closure assay

The cells were grown in 6‐well plates until reaching 80‐90% confluence. A wound was made using a plastic pipette tip across the cell surface. The remaining cells were washed three times to remove any cell debris and incubated at 37°C with serum‐free DMEM. Six different areas of the wound per well were photographed at 0, 12, 24 and 36 hours and the migrating cells were compared. The cell migration distance was determined by measuring the width of the wound and subtracting half of this value from the initial half‐width value of the wound. Each experiment was performed in triplicate and three separate experiments were performed.

### 2.12 Cell transmigration assay

The transmigration assay was conducted in 24‐well transwell chambers (8 μm pore size) using an uncoated membrane. The cells (1 × 10^5^ cells) were suspended in 200 μL serum‐free DMEM and added to the upper chamber, while 500 μL DMEM containing 20% foetal bovine serum was placed in the lower chamber as a chemical attractant. After 24 hours incubation, the cells that did not migrate and remained in the upper chamber were removed by cotton‐tipped swabs. The transmigrated cells that adhered to the bottom surface of the chamber membrane were stained with crystal violet. The cells in ten randomly selected microscopic fields were counted and photographed. The experiment was performed three times in triplicate for each sample (as described above in *Apoptosis detection*).

### 2.13 Plate clone formation

The cells were digested with 0.25% until obtaining single cells and the cell suspension was diluted at a concentration of 1 × 10^4^ cells per mL. Fifteen hundred cells in their medium were added to each well of a six‐well plate and incubated at 37°C under 5% CO_2_. When the clones could be distinguished by naked eye in the six‐well plate, the cell culture stopped, and cells were fixed in 4% paraformaldehyde for 15 minutes. The crystal violet staining was performed and the number of clones of more than 50 cells was counted under the optical microscope. The colony formation rate was calculated as follows: (average number of clones/ number of inoculated cells) × 100%.

### 2.14 Soft agar cloning

Cells were digested, centrifuged, counted and diluted to 1 × 10^4^ cells/mL. Five hundred μL of the agar and 500 μL of the single‐cell suspension was added to each well of a 6‐well plate, and thoroughly mixed. The bottom of the 6‐well plate was covered with a 1.2% agarose layer solidified at room temperature to form a double agar layer. The incubation was performed for 4 weeks at 37°C under 5% CO _2_ with 100 μl complete medium added at intervals to prevent drying. The ell clone formation rate was calculated as follows: Cell clone formation rate = (number of cell clone formation/ number of inoculated cells) × 100%.

### 2.15 Animal experiments

Six‐week‐old BALB/c‐nude mice were obtained from the Experimental Animal Center of Guangxi Medical University (Guangxi, China) and maintained under pathogen‐free conditions. All animal experiments were carried out following the institutional guidelines and were approved by the Committee for Animal Care and Use. Mice were randomly allocated into four groups (293T‐WT, 293T‐KO, HepG2‐WT, HepG2‐KO, n = 6 per group). Cells were resuspended in PBS and 2 × 10^6^ cells resuspended in 40 μL PBS were subcutaneously injected on each side of the forelimb of each nude mouse. Tumours were measured with a calliper, and the volumes were calculated using the equation (length × width 2)/ 2. Thirty‐one days after inoculation, mice were sacrificed and the tumours were excised and subjected to pathological examination. An analytical balance was used to measure the tumour weight, and a Vernier calliper to measure the long diameter as the length and the short diameter as the width, and then the formula (length × width 2)/ 2 was used to calculate the tumour volume.

### 2.16 Statistical analysis

Data analysis, charting and statistical analysis were performed using ImageJ2x, SPSS18.0 and GraphPad Prism 8 software. The comparison was performed using the log‐rank test, chi‐square test, Spearman‐rank correlation test and two‐tailed Student's *t* test. Multivariate statistical analysis was performed using the Cox regression model. Results were expressed as mean ± standard deviation (SD) of triplicates. *P* < 0.05 was considered statistically significant.

## 3 RESULTS

### 3.1 LMNA gene expression in different tumours from HCC patients and different cancer cells

**FIGURE 1:**
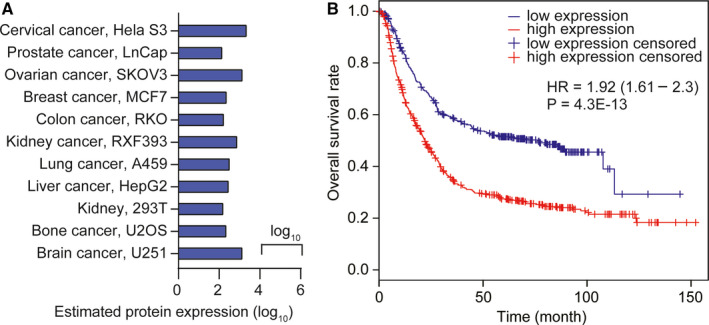
LMNA was up‐regulated in many cancers including HCC, and its high expression was usually related to a lower survival of HCC patients. A, LMNA protein expression in different cancer cell types compared with the correspondent parental cells. LMNA protein expression in normal or tumour tissues and cell lines from Proteomics DB, Max QB and MOPED is shown. B, Kaplan–Meier curve depicting the survival of HCC patients with high or low LMNA expression

To explore the specific changes in the expression of the LMNA gene in various tumours, a comparative analysis was performed between normal tissues and corresponding tumours as well as on different types of cancer cells using data obtained from the Proteomics DB, Max QB and MOPED databases. The data revealed that LMNA expression was significantly up‐regulated in brain cancer cells (U251), bone cancer cells (U2OS), kidney cells (293T) and liver cancer cells (HepG2) (Figure 1A). The Kaplan–Meier curve of patients with HCC showed a lower survival rate in patients with high LMNA expression (Figure 1B).

### 3.2 LMNA knockout cell lines from 293T and HepG2 by CRISPR/Cas9 technology

**FIGURE 2:**
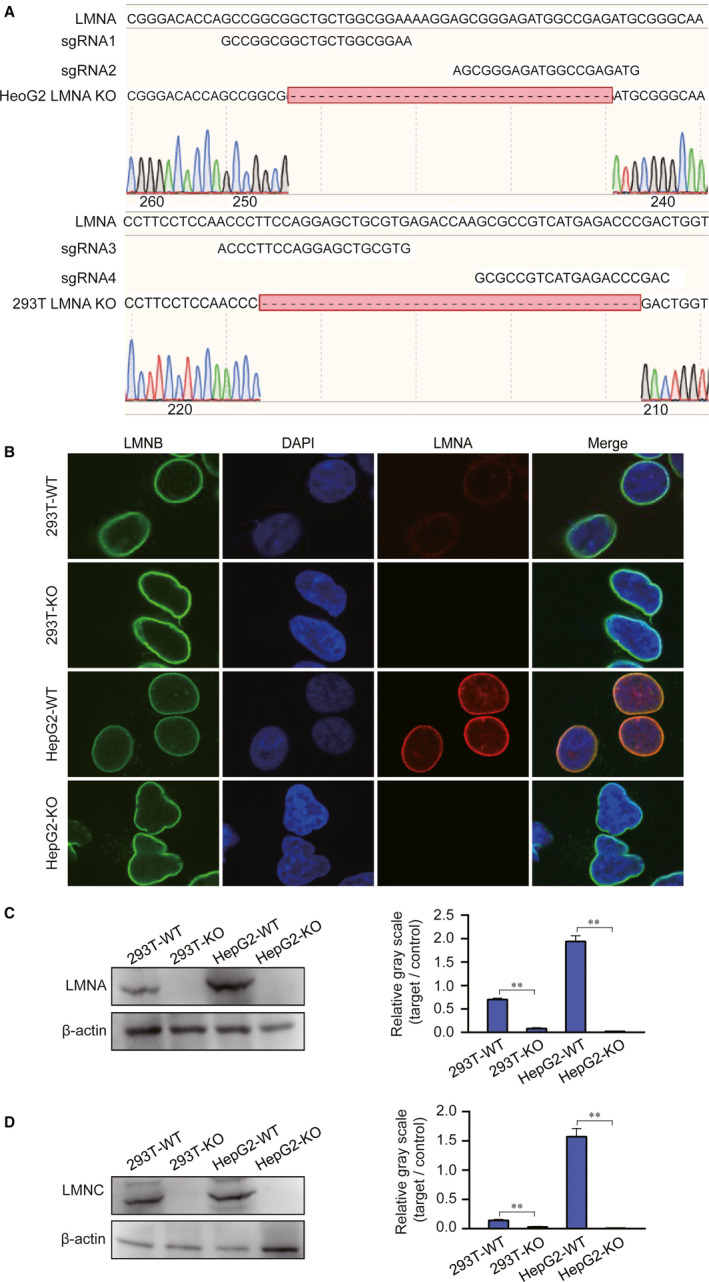
Knockout of the LMNA gene in 293T and HepG2 cells by the CRISPR/Cas9 system. A, The recognition site of the gRNAs used for the two cell lines in the CRISPR/Cas9 system. B, Immunofluorescence assay to detect the expression of the LMNA protein. No LMNA (red) signal appeared in LMNA knockout cell lines. C and D, Detection of Lamin A/C by western blot in WT and knockout cell lines. KO, LMNA knockout; WT, wild‐type. (**P < .01)

To further study the function of the LMNA gene, LMNA knockout cell lines were acquired by the CRISPR/Cas9 technology (Figure 2A). Immunofluorescence and western blot were performed to verify whether the LMNA gene was successfully knocked out. The immunofluorescence results revealed that both cell lines did not express the LMNA protein, while the LMNB protein expression was unaffected (Figure 2B). The WB results confirmed this result (Figure 2C). Besides, the LMNC protein which is translated by the same LMNA gene was also down‐regulated (Figure 2D).

### 3.3 LMNA knockout attenuated cell proliferation, arrested cell cycle and increased cell apoptosis in 293T and HepG2 cell lines

**FIGURE 3:**
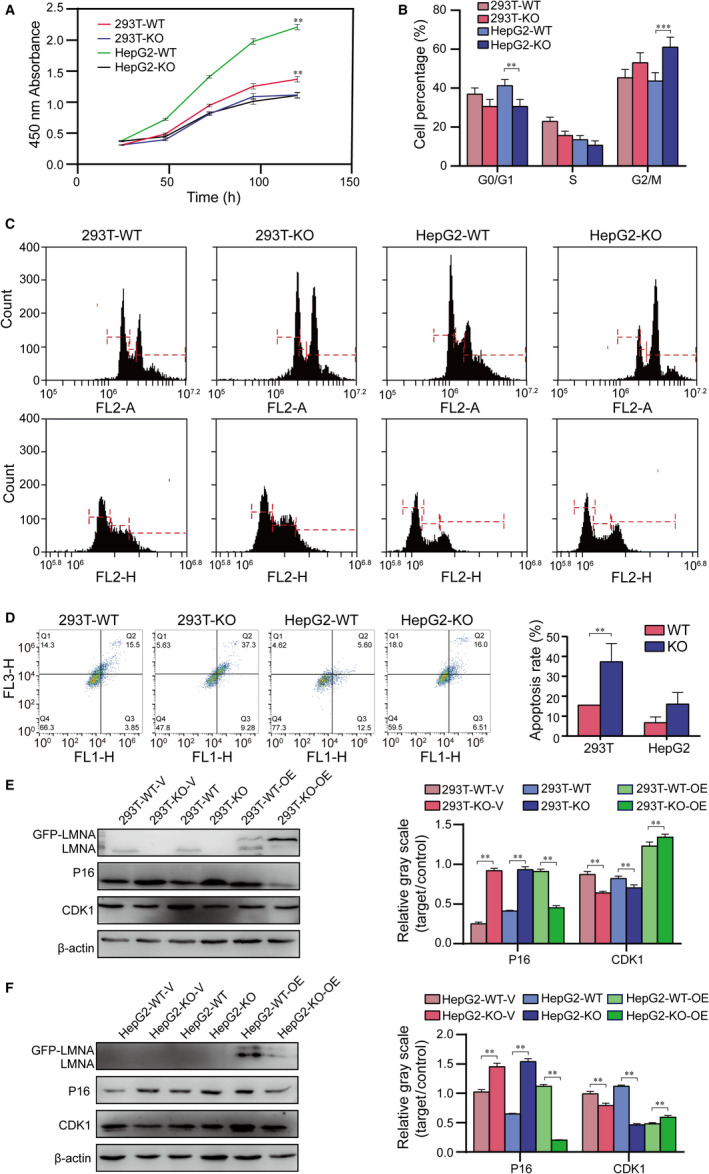
Effect of LMNA knockout on cell proliferation and apoptosis in HepG2 and 293T cells. A, Proliferation of wild‐type and LMNA knockout cell lines by CCK8 assay. Both 293T and HepG2 knockout cell lines showed a decreased cell proliferation. B and C, FACS cell cycle analysis revealed that HepG2‐knockout cells underwent G2/M phase arrest. D, Cell apoptosis by Annexin V and PI, and analysed by FACS. Both 293T and HepG2 knockout cell lines underwent higher rate of late apoptosis. E, Western blot results of P16 and CDK1 protein in 293T cell line and correspondent quantification. F, Western blot results of P16 and CDK1 protein in HepG2 cell line and correspondent quantification. Results were expressed as mean ± SD, calculated from three independent experiments. (*P < .05, **P < .01). WT: wild‐type; KO: LMNA knockout; WT + V: wild‐type plus blank vector; KO + V: LMNA knockout plus blank vector; WT + OE: wild‐type plus exogenous expression of LMNA; KO + OE: LMNA knockout plus exogenous expression of LMNA

Next, a series of function experiments were performed using the knockout cell lines to elucidate the LMNA gene function. The results showed that the proliferation of both LMNA knockout cell lines decreased (*P* < 0.01), and the decrease of HepG2‐knockout cells was more significant (Figure 3A). HepG2‐knockout cells underwent G2/M phase arrest (Figure 3B and C) and the cell apoptosis rate (Figure 3D) significantly increased (*P* < 0.05 in both LMNA knockout cell lines).

Moreover, western blot was used to detect the expression of P16, which is a suppressor of the cell cycle, and CDK1, which is a marker of the G2/M phase. P16 expression was up‐regulated and CDK1 expression was down‐regulated in 293T‐knockout and HepG2‐knockout cell lines compared with the wild‐type cells. The exogenous expression of GFP‐LMNA in each group of cells (the methods were mentioned above, in the *CRISPR/Cas9 technique*) resulted in a decreased P16 expression and increased CDK1 expression (Figure 3E and F). Therefore, it was proved that the loss of LMNA gene expression resulted in the up‐regulation of P16 and down‐regulation of CDK1, suggesting that the loss of the LMNA gene caused the proliferation and cell cycle arrest of the tumour cells.

### 3.4 Knockout of the LMNA gene in 293T and HepG2 cells led to a decreased cell migration and colony formation, and improved the transmigration ability

**FIGURE 4:**
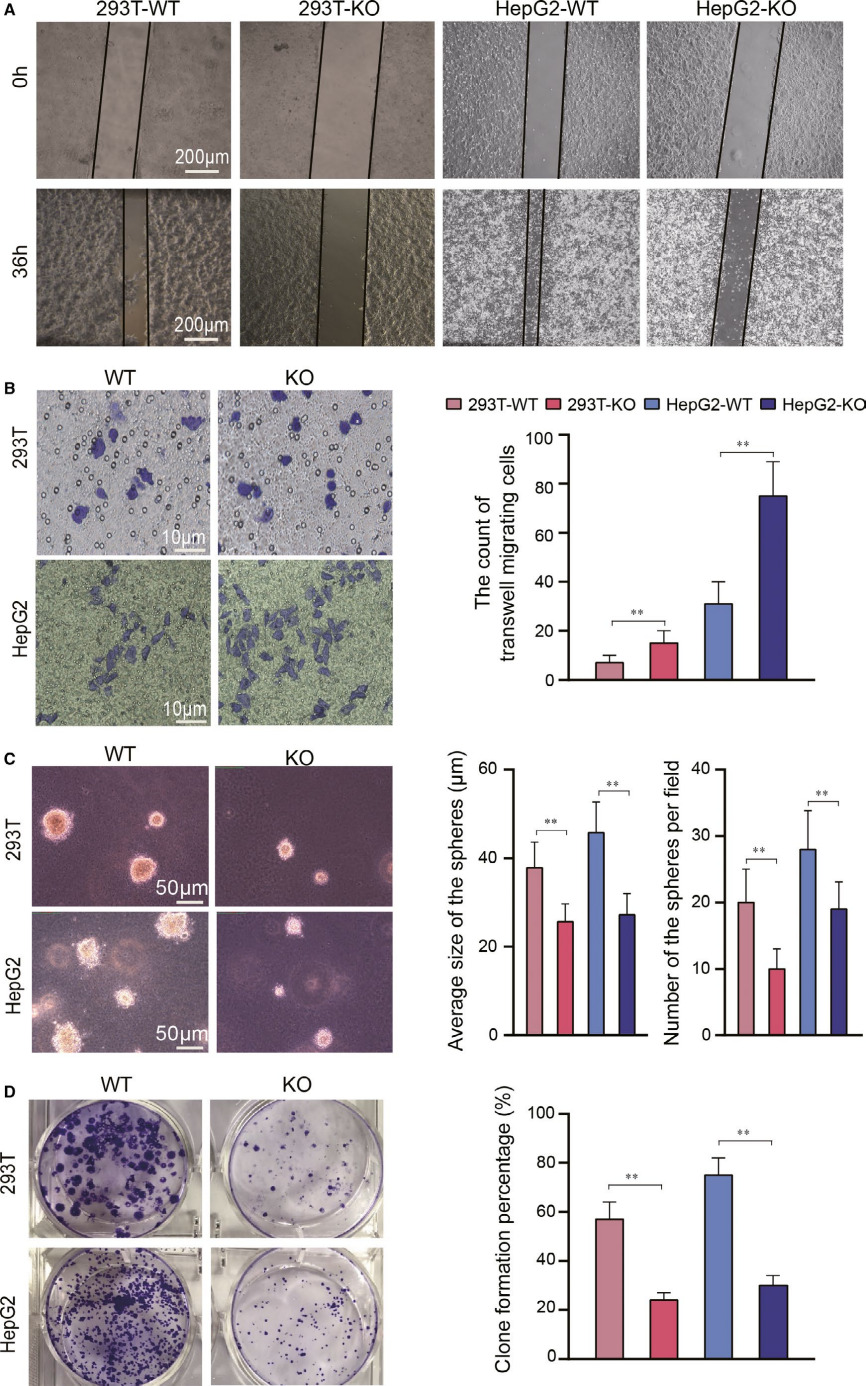
Influence of LMNA knockout on 293T and HepG2 cell migration, transmigration and ability of clone formation. Knockout of LMNA gene in 293T and HepG2 cells leads to (A) a decreased wound closure ability, and (B) increased cell transmigration ability (C) decreased formation of soft agar clones and (D) decreased platelet colony formation. Soft agar colony formation of the two LMNA knockout cells resulting in a decrease in the cloning rate and the size of the clone. Results were expressed as mean ± SD of triplicates (**P < .01)

After the function tests, the tumorigenicity of the LMNA knockout cell lines was evaluated in vitro. The wound closure assay showed that the healing ability of both LMNA knockout cell lines was significantly decreased (Figure 4A). In contrast, the transwell migration tests showed that the transmigration ability of both LMNA knockout cell lines was enhanced (Figure 4B). Furthermore, the colony formation ability of each group of cells showed that the colony formation rate (Figure 4C) and the average size of the clones (Figure 4D) of the two LMNA knockout cell lines decreased (both *P* < 0.01). The migration ability of 293T and HepG2 cells decreased in vitro after LMNA knockout and the tumorigenicity ability as well. These results suggested that knockout of the LMNA gene in 293T and HepG2 cells led to a decreased cell migration and colony formation, and improved the transmigration ability of the cell lines.

### 3.5 *LMNA knockout decreased the xenograft tumour growth* in vivo

**FIGURE 5:**
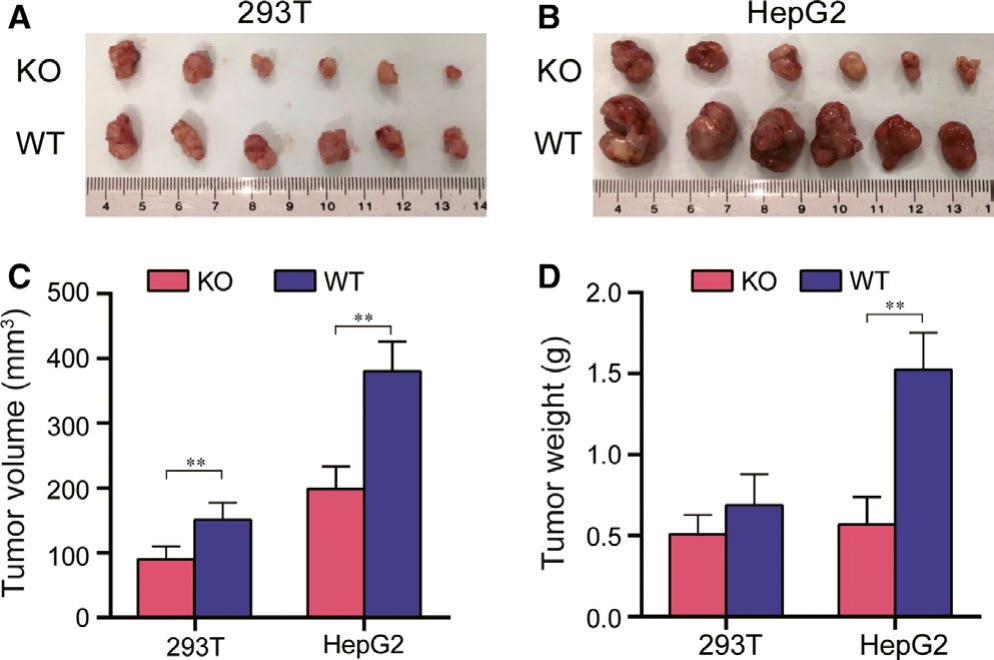
Effect of LMNA knockout on the tumorigenicity in a nude mouse xenograft model. A, Tumorigenic analysis of 293T WT and knockout cells (2 × 10^6^ cells for each group) in nude mice (n = 6) for 30 days. B, Analysis of the tumour formed by HepG2 WT and knockout cells (2 × 10^6^ cells for each group) in nude mice (n = 6) for 30 days. C, Volume of the subcutaneous tumours by wild‐type and LMNA knockout cell lines in nude mice (293T group *P < .05). D, Weight of the subcutaneous tumours by wild‐type and LMNA knockout cell lines in nude mice. Results were expressed as mean ± SD of triplicates (*P < .05, **P < .01)

After discovering the changes in the tumorigenic ability of LMNA knockout cells in vitro, the tumorigenic ability of HepG2 and 293T LMNA knockout cell lines in nude mice was investigated. The subcutaneous tumours in nude mice were smaller in volume (293T, *P* < 0.05) and weight (HepG2, *P* < 0.05) when the two LMNA knockout cells were subcutaneously injected in nude mice compared with the volume and weight when the corresponding WT cells were injected (Figure 5A–D).

### 3.6 WB analysis indicated that the ECM and cancer signalling pathway was changed after LMNA knockout

**FIGURE 6:**
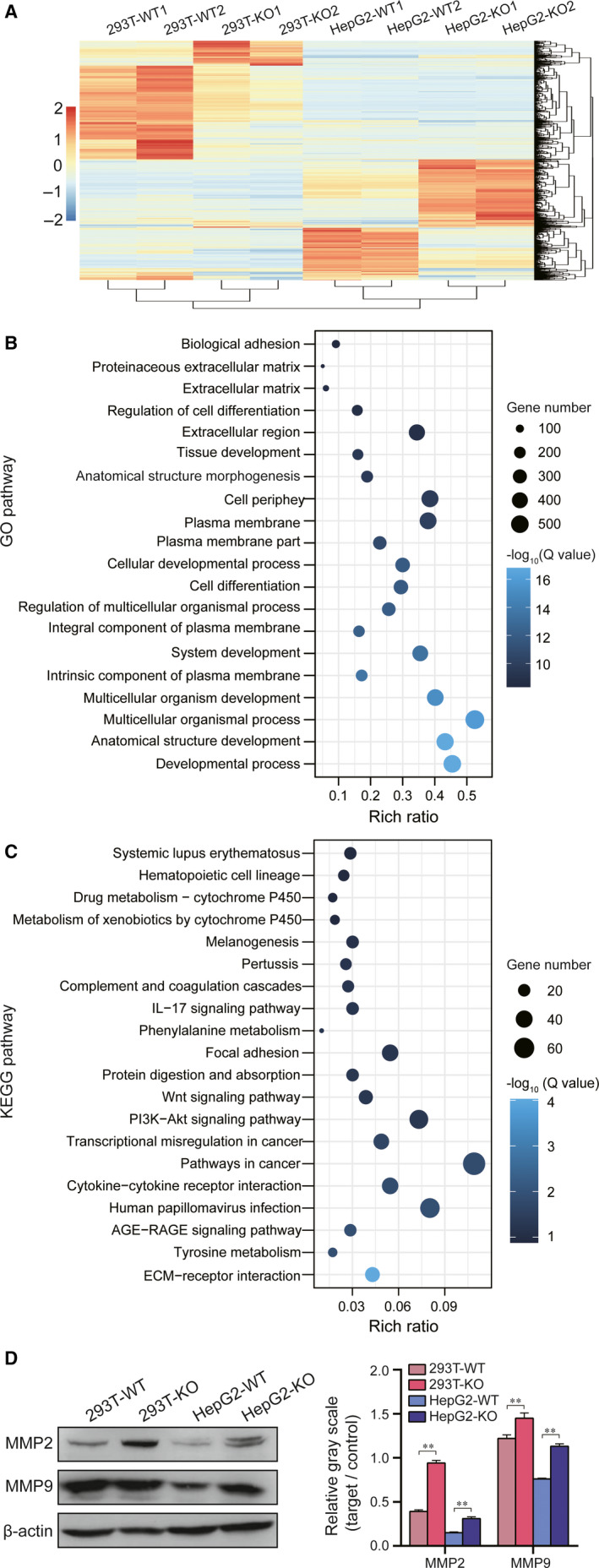
Differentially expressed genes in the LMNA knockout cell lines and their correspondent wild‐type by RNA‐seq. A, Heat map of 8 samples; the up‐regulated genes are shown in red colour, and the down‐regulated genes are shown in blue colour, the deeper the colour, the bigger the expression difference. The scale was by log_10_. B, GO analysis of the differential gene sets in the wild‐type and LMNA knockout cell lines (WT *vs* KO). C, KEGG pathway analysis of differential gene sets in the wild‐type and LMNA knockout cell lines (WT vs KO). D, Western blot results of MMP2/9 protein expression. Results were expressed as mean ± SD of triplicates (**P < .01)

After concluding that the LMNA gene knockout resulted in a decrease in the tumorigenic capacity of tumour cells, the relevant molecular mechanism was investigated. RNA‐seq analysis of the LMNA knockout cell lines and wild‐type cells, which were obtained by the CRISPR/Cas9 technique described above, was performed, and the results showed that two different batches of four cells could be clustered (Figure 6A). In addition, the GO enrichment map (Figure 6B) and KEGG pathway (Figure 6C) enrichment map showed the presence of many differentially expressed genes and regulatory pathways in LMNA knockout cells and wild‐type cells. The expression of MMP2 and MMP9 in the ECM signalling pathway was then evaluated by WB, and the result showed that the expression of MMP2 and MMP9 was decreased (*P* < 0.01) in the two LMNA knockout cell lines (Figure 6D), which was consistent with the RNA‐seq results.

## 4 DISCUSSION

The role of LMNA gene in tumours, in the development and progression of HCC and its molecular mechanism is still a challenge. In the current study, the relationship between LMNA and HCC was evaluated. Our results of the Kaplan–Meier survival analysis from the data of 876 HCC patients stored in the databases revealed that a lower survival was associated to a high LMNA expression. In addition, the two LMNA knockout cell lines showed a decreased tumorigenicity in vivo and in vitro, an up‐regulated expression of P16, and a down‐regulated expression of CDK1. The overexpression of the LMNA gene in the knockout cells was associated with a decreased P16 expression and an increased CDK1 expression. Combined with the clinical data, our results demonstrated that the LMNA gene might work as an oncogene in HCC. Our study successfully linked the LMNA gene expression and the expression of P16 and CDK1 in HepG2 and 293T cell lines, providing a basis for exploring the relationship between LMNA gene and tumorigenesis in various tumour types. In addition, our discovery might provide a potential new target for the diagnosis and treatment of HCC.

In this study, our hypothesis was that LMNA might play an oncogene role in HCC since HCC patients with higher LMNA expression showed a lower survival rate according to the Kaplan–Meier curve. It is well known that the most important pathological type of HCC is the primary liver cancer, which accounts for approximately 90%.^17^, ^18^ LMNB1 expression (lamin B) is significantly up‐regulated in HCC patients, thus, its expression may be used as a prognostic indicator in patients at an early‐ and late‐stage HCC.^19^ Lamin A, a nuclear lamina structural protein like lamin B, is critical for the stabilization of retinoblastoma tumour suppressor proteins pRb and p107.^20^, ^21^, ^22^ These discoveries suggest that Lamin A/B might be closely related to the tumorigenesis.

In this work, LMNA protein expression in HepG2, and cells was significantly up‐regulated suggesting that the LMNA gene might be relate to the malignant degree of tumour cells. In addition, the proliferation ability of HepG2 cells decreased after LMNA knockout and the cell cycle was arrested. Previous studies showed that the knock down of lamin A/C in human lung cancer cell lines leads to an increased tumour growth rate *in vivo*.^21^, ^23^ However, the knock down of lamin A/C in human primary diploid fibroblasts leads to G1 arrest and inhibits cell proliferation.^24^ Thus, our conclusion was that the knockout of the LMNA gene in different cells has a different effect on cell proliferation and cell cycle, thus potentially explaining the different role of LMNA in different tumours.

In this study, we also found that P16 expression increased after knockout of the LMNA in HepG2 cells. P16 expression significantly decreased after the overexpression of LMNA, indicating that the LMNA gene could regulate the expression of P16. Subsequent experiments of tumour formation in nude mice also demonstrated that LMMA expression promoted tumour growth while LMNA knockout inhibited tumour growth. As a tumour suppressor gene, P16 is inactivated in various tumours, such as oropharyngeal cancer,^25^, ^26^, ^27^ breast cancer^28^, ^29^, ^30^ and pancreatic adenocarcinoma,^31^, ^32^ and it is closely relates to the occurrence and development of tumours. Therefore, LMNA gene expression in HepG2 cells may suppress the P16 function and promote tumorigenesis.

The molecular mechanism was further investigated using western blot. Our results showed that the expression of MMP2/9 in LMNA knockout cells was up‐regulated after the knockout of the LMNA gene in 293 and HepG cells. MMPs can degrade the extracellular matrix, leading to the removal of the tumour invasion barrier, making easier for tumour cells the invasion of adjacent tissues.^33^, ^34^, ^35^ The two LMNA knockout cell lines showed enhanced transmigration ability. The decrease in lamins potentiates cancer cell migration through narrow spaces, suggesting a potential role in metastasis.^23^, ^36^, ^37^, ^38^ In our study, the migration ability of the two LMNA knockout cell lines was significantly lower than that of the wild‐type cells, which is consistent with previous findings^23^, ^36^, ^38^. On the contrary, the transwell migration ability increased in knockout cell lines. Our hypothesis was that the loss of lamin A/ C might result in a thinner nuclear membrane and an easier ability of deformation to pass through narrow gaps; furthermore, the loss of LMNA up‐regulated the expression of the MMPs, making tumour cells more prone to invasion and metastasis. Knockout cells have various residual lamin A/ C expression, which has a certain impact on LMNA gene functional studies. Compared with previous LMNA gene knockdown experiments, knockout cell lines could provide more convincing evidence of functional study.

**FIGURE 7:**
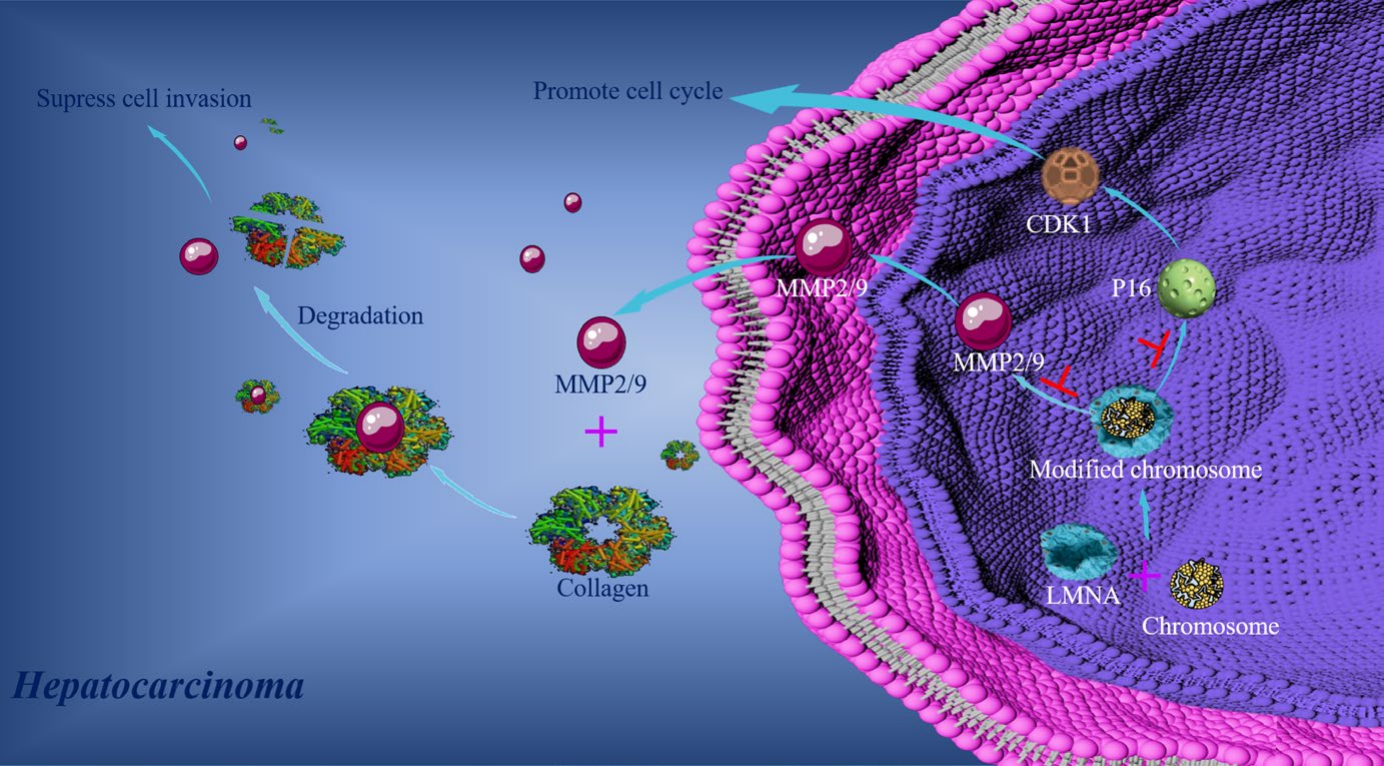
Model of LMNA gene regulating the migration and proliferation of HCC cells HepG2. LMNA binding to the chromosome resulted in the down‐regulation of P16 and MMP2/9, then the collagen degradation was inhibited and the expression of CDK1 was up‐regulated. Finally, cell proliferation was promoted and cell invasion was suppressed

Taken together, the results in the current study, a model illustrating the LMNA gene regulation of the migration and proliferation of HCC cells HepG2 might be proposed (Figure 7). On one hand, LMNA inhibits the expression of MMP2/9, resulting in the decrease of the extracellular matrix degradation and the suppression of cell invasion. On the other hand, LMNA down‐regulates the expression of P16 (the tumour suppressor gene), resulting in the up‐regulation of CDK1. Our hypothesis was that molecular mechanism of the interaction between LMNA and MMP2/9, or between LMNA and P16/CDK1 might provide new insight in HCC development, as well as new drug targets. Therefore, the LMNA gene might represent a novel biomarker to evaluate the malignant degree of HCC.

## CONFLICT OF INTEREST

The authors confirm that there are no conflicts of interest.

## AUTHORS CONTRIBUTIONS

Heng Liu: Data curation (lead); Formal analysis (lead); Investigation (lead). Dongming Li: Data curation (equal); Formal analysis (equal); Investigation (equal). Ling Zhou: Data curation (equal); Investigation (equal). Shuang Kan: Data curation (equal); Investigation (equal). Guozhang He: Data curation (equal); Investigation (equal). Kun Zhou: Data curation (equal); Formal analysis (equal). Liping Wang: Conceptualization (equal); Supervision (equal); Writing‐original draft (lead); Writing‐review & editing (lead). Ming Chen: Conceptualization (equal); Writing‐original draft (equal); Writing‐review & editing (equal). Wei Shu: Conceptualization (lead); Supervision (lead); Writing‐original draft (equal); Writing‐review & editing (equal).

## Data Availability

The data that support the findings of this study are available from the corresponding author upon reasonable request.
